# Correlation analysis of hepatic steatosis and hepatitis B virus: a cross-sectional study

**DOI:** 10.1186/s12985-023-02277-8

**Published:** 2024-01-19

**Authors:** Sitong Yi, Guanghui Ren, Ying Zhu, Qingwei Cong

**Affiliations:** Department of Infectious Disease and Liver Disease Center of Integrated Traditional Chinese and Western Medicine, The First Affiliated Hospital of Dalian Medical University, Dalian, Liaoning China

**Keywords:** Hepatic steatosis, Hepatitis B virus, MAFLD, CHB, pgRNA

## Abstract

**Background:**

The co-occurrence of chronic hepatitis B (CHB) and metabolic dysfunction-associated fatty liver disease (MAFLD) has drawn considerable attention due to its impact on disease outcomes. This study aimed to investigate the association between hepatic steatosis and hepatitis B virus (HBV) and analyzed the influence of hepatic steatosis on hepatitis B virology in patients with CHB.

**Methods:**

In this cross-sectional study, 272 patients infected with HBV who were treatment-naïve or had ceased antiviral treatment for > 6 months were categorized into the CHB group (n = 128) and CHB + MAFLD group (n = 144). Furthermore, based on whether HBV DNA was higher than 2000 IU/mL, patients were categorized into the high-level HBV DNA group (n = 129) and the low-level HBV DNA group (n = 143). The impact of hepatic steatosis on hepatitis B virology was analyzed within the CHB cohort. Multivariate logistic regression analysis was employed to identify independent factors influencing pre-genomic RNA (pgRNA) levels below the lower limit of detection (LLD) in patients with CHB.

**Results:**

Among the 272 patients, compared with CHB group, HBV DNA levels (4.11 vs. 3.62 log_10_ IU/mL, *P* = 0.045), hepatitis B surface antigen (HBsAg) levels (3.52 vs. 3.20 log_10_ IU/mL, *P* = 0.008) and the hepatitis B e antigen (HBeAg) positive rate (33.6% vs. 22.2%, *P* = 0.036) were significantly decreased in the CHB + MAFLD group; In 143 low-level HBV DNA patients, the CHB + MAFLD group exhibited decreased levels of pgRNA and HBsAg compared to the CHB group. However, in 129 high-level HBV DNA patients, a more significant decrease was observed in pgRNA (3.85 vs 3.35 log_10_ copies/mL, *P* = 0.044) and HBsAg (3.85 vs 3.59 log_10_ IU/mL, *P* = 0.033); Spearman correlation analysis revealed a negative correlation between hepatic steatosis and pgRNA (r =  − 0.529, *P* < 0.001), HBV DNA (r =  − 0.456, *P* < 0.001), HBsAg (r =  − 0.465, *P* < 0.001) and HBeAg (r =  − 0.339, *P* < 0.001) levels; Multivariate logistic regression analysis identified HBV DNA (odds ratio [OR] = 0.283, *P* < 0.001), HBsAg (OR = 0.300, *P* < 0.001), and controlled attenuation parameter (CAP) values (OR = 1.013, *P* = 0.038) as independent factors influencing pgRNA levels below the LLD in patients with CHB.

**Conclusions:**

This study establishes a negative correlation between hepatic steatosis and hepatitis B virology, demonstrating decreased HBV expression in patients with CHB + MAFLD.

## Introduction

Hepatitis B virus (HBV) and metabolic dysfunction-associated fatty liver disease (MAFLD) are prevalent chronic liver diseases with the potential to progress to liver fibrosis, cirrhosis, and hepatocellular carcinoma (HCC), significantly impacting patient prognosis. The coexistence of these conditions in many patients can further influence disease evolution and prognosis.

The pathogenesis of the coexistence of chronic hepatitis B (CHB) and MAFLD is intricate. Numerous studies have aimed to elucidate their interaction, marking a challenging and prominent research focus in chronic liver disease [1, 2]. An animal study indicated that hepatic steatosis attenuated the expression of HBV DNA, hepatitis B surface antigen (HBsAg), and pre-genomic RNA (pgRNA) in immunocompetent mice, inhibiting HBV replication [3]. This inhibition could potentially benefit achieving clinical cure in patients with CHB. However, some scholars argue that MAFLD-related lipid metabolism disorders and lipotoxicity may induce inflammation, disrupting hepatocyte homeostasis, resulting in elevated alanine aminotransferase (ALT) levels in patients with CHB, with potential progression to cirrhosis and HCC [4]. Consequently, further investigation into the role of hepatic steatosis in HBV infection is warranted. Recent research suggests that the true virological response requires HBV DNA below detection limits, considering the recognized replication cycle of HBV infection. HBV DNA disappearance indicates the inhibition of reverse transcription and not that of covalently closed circular DNA (cccDNA) transcription. A comprehensive virological response necessitates both HBV DNA and HBV RNA to be below the lower limit of detection (LLD) [5]. As a precursor of HBV RNA, pgRNA authentically reflects cccDNA transcriptional activity in hepatocytes. Therefore, serum pgRNA levels can reflect HBV replication in the hepatocytes of patients. In summary, this study investigated hepatic steatosis and hepatitis B virology, explored the influencing factors of pgRNA below LLD in patients infected with HBV, and delved into the potential impact of liver steatosis on HBV replication ability.

## Materials and methods

### Study population

This cross-sectional study involved 272 patients with chronic HBV infection who visited the Liver Disease Center of Integrated Traditional Chinese and Western Medicine at Dalian Medical University between July 2021 and November 2023. All patients were either treatment-naïve or had ceased antiviral treatment for > 6 months. Based on the presence of fatty liver, patients were categorized into the CHB group (CHB) with 128 cases, and the CHB combined with the MAFLD group (CHB + MAFLD) with 144 patients. Further classification into high-level HBV DNA (HBV DNA ≥ 2000 IU/mL) (n = 129) and low-level HBV DNA (HBV DNA < 2000 IU/mL) (n = 143) groups was done based on whether HBV DNA was higher than 2000 IU/mL. Patient data encompassing general conditions were collected, and various examinations, including serum biochemistry, HBV serology, HBV DNA quantification, pgRNA quantification, and FibroScan examination, were conducted. Serum samples were collected and stored at − 80°C.

The inclusion criteria were as follows: (1) age ≥ 18 years; (2) Positive HBsAg, or HBV DNA, or both for > 6 months; (3) Diagnosis of MAFLD met the criteria outlined in “A new definition for metabolic dysfunction-associated fatty liver disease: An international expert consensus statement” [6]. Exclusion criteria included: (1) Concurrent presence of other viral hepatitis; (2) Coexistence with other liver diseases such as autoimmune liver disease or drug-induced liver injury; (3) Incomplete clinical data collection.

### Serum pgRNA and HBV DNA detection

Serum pgRNA was assessed using RNA simultaneous amplification testing (SAT) with the SAT isothermal amplification real-time fluorescence detection kit (Rendu Biotechnology, Shanghai, China). The Auto SAT system (Rendu Biotechnology) facilitated RNA extraction, amplification, and detection. The linear concentration range covered 2 log copies/mL to 8 log copies/mL, with a 50 copies/mL LLD.

Serum HBV DNA levels were determined through real-time polymerase chain reaction utilizing the HBV nucleic acid quantitative detection kit (Shengxiang Biotechnology, Hunan, China), with a reference range of HBV DNA < 20 IU/mL.

### HBV serology detection

Serum HBsAg was detected by Abbott Microparticle chemiluminescence i2000 (Abbott, Chicago, USA), with a reference range set at HBsAg > 0.05 IU/mL. HBeAg levels were assessed using a Roche e801 automatic electrochemiluminescence immunoassay analyzer (Roche, Basel, Switzerland), and the reference range was < 1.0 signal-to-cutoff ratio [S/Co].

### FibroScan assessment

For the evaluation of the controlled attenuation parameter (CAP) and liver stiffness measurement (LSM), the FibroScan®502 and M-type probe (Echosens, Paris, France) were utilized. The procedure required successful execution ten times at the same position, and the median of the effective measurement results was considered the outcome. Following the meta-analysis published in 2017 [7], CAP values were categorized as follows: no steatosis (grade 0 [S0]) with CAP < 248 dB/M; mild steatosis (grade 1 [S1]) with 248 dB/M ≤ CAP < 268 dB/M; moderate steatosis (grade 2 [S2]) with 268 dB/M ≤ CAP < 280 dB/M; and severe hepatic steatosis (grade 3 [S3]) with CAP ≥ 280 dB/M.

### Statistical analysis

Continuous variables were expressed as median (interquartile range [IQR]), and group comparisons were conducted using the Mann‒Whitney U test or Kruskal–Wallis test. Categorical variables were presented as percentages (%), and inter-group differences were assessed using the Chi-squared and Fisher’s exact tests. One-way analysis of variance detected variations in HBV levels among groups with varying hepatic steatosis. Spearman correlation coefficient was employed to assess the correlation between hepatic steatosis and hepatitis B virology. Univariate and multivariate logistic regression analyses were performed to identify factors influencing pgRNA below the LLD, and odds ratio (OR) with 95% confidence intervals (95% CI) were calculated. Statistical significance was set at *P* < 0.05. Statistical analysis was conducted using IBM SPSS (Version 25.0).

## Results

### Clinical characteristics of 272 patients

Throughout the study, 272 patients were enrolled, and Table 1 summarizes their clinical profiles. The cohort comprised 156 men (57.4%) and 116 women (42.6%), with a median age of 44 years. Among them, 138 cases (50.7%) had a family history of HBV, 31 (11.4%) had diabetes mellitus, 34 (12.5%) had cirrhosis, and 14 (5.1%) had HCC. The distribution of hepatic steatosis was as follows: 128 cases without hepatic steatosis, 51 with S1, 41 with S2, and 52 with S3.

**Table 1:** Clinical profiles of 272 patients with Hepatitis B infection

Variables	CHB (n = 128)	CHB + MAFLD (n = 144)	Total (n = 272)	*P* value
Male, n (%)	64 (50.0)	92 (63.9)	156 (57.4)	0.021*
Age (years)	47 (36–54)	44 (38–53)	44 (37–53)	0.898
Family history of HBV, n (%)	65 (50.8)	73 (50.7)	138 (50.7)	0.989
Diabetes, n (%)	8 (6.3)	23 (16.0)	31 (11.4)	0.012*
Cirrhosis, n (%)	10 (7.8)	24 (16.7)	34 (12.5)	0.028*
Hepatocellular carcinoma, n (%)	7 (5.5)	7 (4.9)	14 (5.1)	0.821
BMI (kg/m^2^)	23.9 (22.1–25.3)	25.1 (24.2–26.5)	24.6 (23.1–26.0)	< 0.001*
Overweight/obesity^a^, n (%)	54 (42.2)	113 (78.5)	167 (61.4)	< 0.001*
Waist-to-hip ratio	0.88 (0.83–0.92)	0.89 (0.84–0.96)	0.89 (0.83–0.95)	0.142
CAP values (dB/m)	209 (186–227)	273 (259–295)	250 (214–274)	< 0.001*
LSM values (kPa)	5.5 (4.5–7.8)	6.7 (5.1–8.5)	6.2 (4.6–8.1)	0.233
ALT (U/L)	28.0 (19.3–37.0)	29.0 (21.3–43.0)	29.0 (21.0–39.0)	0.708
Elevated ALT^b^, n (%)	22 (17.2)	41 (28.5)	63 (23.2)	0.028^*^

Data are expressed as median (IQR) or number (%)

*Statistically significant at *P* < 0.05

*BMI* Body mass index, *CAP* Controlled attenuation parameter, *LSM* Liver stiffness measurement, *ALT* Alanine aminotransferase

^a^The study included Chinese participants, with overweight/obesity defined as BMI ≥ 24 kg/m^2^

^b^Elevated ALT was considered when exceeding the local upper limit of normal (ULN), with ALT > 40 U/L

Significant differences were observed between the CHB + MAFLD and CHB groups in terms of male proportion, history of diabetes mellitus, history of cirrhosis, body mass index (BMI), ratio of overweight/obesity individuals, CAP values, and proportion of elevated ALT (*P* < 0.05). However, the two groups showed no significant differences in age, family history of HBV, history of HCC, waist-to-hip ratio, LSM values, and ALT levels.

### Association between hepatic steatosis and hepatitis B virology

#### *Comparative analysis of CHB and CHB* + *MAFLD in 272 patients infected with HBV*

In the cohort of 272 cases with HBV infection, CHB + MAFLD exhibited an increased proportion of HBV DNA and pgRNA below the LLD compared to CHB (HBV DNA: 39.6% vs. 29.7%; pgRNA: 27.8% vs. 18.8%, respectively). Moreover, the levels of HBV DNA (4.11 vs. 3.62 log_10_ IU/mL, *P* = 0.045) and HBsAg (3.53 vs. 3.15 log_10_ IU/mL, *P* = 0.008) were significantly decreased in CHB + MAFLD, along with a lower positive rate of HBeAg (33.6% vs. 22.2%, *P* = 0.036) (Table 2).

**Table 2:** Comparative analysis of hepatitis B virology in 272 patients with CHB and CHB + MAFLD

Variables	N	CHB (n = 128)	CHB + MAFLD (n = 144)	*P* value
HBV DNA below the LLD, n (%)	95	38 (29.7)	57 (39.6)	0.088
HBV DNA (log_10_ IU/mL)	177	4.11 (3.32–5.17)	3.62 (3.08–4.48)	0.045*
pgRNA below the LLD, n (%)	64	24 (18.8)	40 (27.8)	0.080
pgRNA (log_10_ copies/mL)	208	3.52 (2.99–4.24)	3.20 (2.63–3.85)	0.084
HBsAg (log_10_ IU/mL)	272	3.53 (2.71–3.97)	3.15 (2.17–3.55)	0.008*
Positive rate of HBeAg, n (%)	75	43 (33.6)	32 (22.2)	0.036*

Data are expressed as median (IQR) or number (%)

*Statistically significant at *P* < 0.05

#### *Comparative analysis of hepatitis B virology in the high-level HBV DNA cohort with CHB and CHB* + *MAFLD*

In 129 high-level HBV DNA patients, compared with CHB group, the levels of pgRNA (3.85 vs. 3.35 log10 copies/mL, *P* = 0.044) and HBsAg (3.85 vs. 3.59 log10 IU/mL, *P* = 0.033) were significantly decreased in CHB + MAFLD group. Furthermore, the positive rate of HBeAg was lower in CHB + MAFLD group (Table 3).

**Table 3:** Comparative analysis of hepatitis B virology in high-level HBV DNA patients with CHB and CHB + MAFLD

Variables	N	CHB (n = 68)	CHB + MAFLD (n = 61)	*P* value
pgRNA below the LLD, n (%)	5	2 (2.9)	3 (4.9)	0.561
pgRNA (log_10_ copies/mL)	124	3.85 (3.09–4.73)	3.35 (2.88–3.93)	0.044*
HBsAg (log_10_ IU/mL)	129	3.85 (3.43–4.21)	3.59 (3.23–3.95)	0.033*
Positive rate of HBeAg, n (%)	60	34 (50.0)	26 (42.6)	0.402

Data are expressed as median (IQR) or number (%)

*Statistically significant at *P* < 0.05

#### *Comparative analysis of hepatitis B virology in the low-level HBV DNA cohort with CHB and CHB* + *MAFLD*

In 143 low-level HBV DNA patients, CHB + MAFLD showed an increase in the proportion of pgRNA below the LLD (36.7% vs. 44.6%, respectively). Furthermore, the levels of pgRNA (3.09 vs. 2.96 log10 copies/ml, respectively), HBsAg (2.74 vs. 2.69 log10 IU/ml, respectively) and the positive rate of HBeAg (15.0% vs. 7.2%, respectively) were decreased in CHB + MAFLD (Table 4).

**Table 4:** Comparative analysis of hepatitis B virology in low-level HBV DNA patients with CHB and CHB + MAFLD

Variables	N	CHB (n = 60)	CHB + MAFLD (n = 83)	*P* value
pgRNA below the LLD, n (%)	59	22 (36.7)	37 (44.6)	0.343
pgRNA (log_10_ copies/mL)	84	3.09 (1.88–3.62)	2.96 (2.28–3.40)	0.593
HBsAg (log_10_ IU/mL)	143	2.74 (1.35–3.62)	2.69 (1.68–3.17)	0.279
Positive rate of HBeAg, n (%)	15	9 (15.0)	6 (7.2)	0.134

Data are expressed as median (IQR) or number (%)

### Correlation analysis between hepatic steatosis and hepatitis B virology

**Fig. 1:**
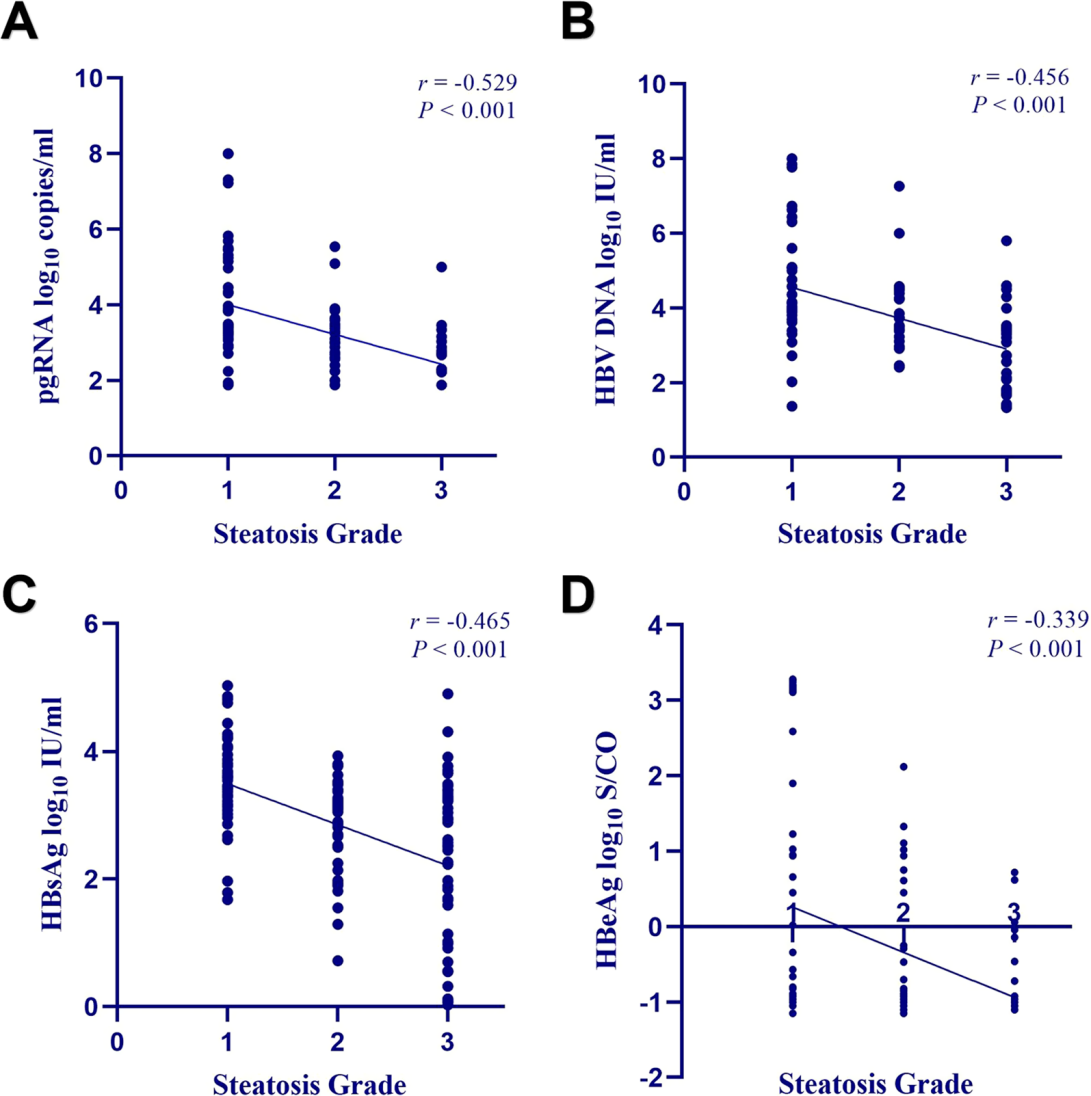
Correlation between hepatic steatosis and hepatitis B virology in 144 patients with CHB + MAFLD. **A** pgRNA, **B** HBV DNA, **C** HBsAg, and **D** HBeAg were negatively correlated with the degree of hepatic steatosis

A correlation analysis investigating the relationship between the severity of liver steatosis and hepatitis B virology was performed on 144 patients with CHB + MAFLD. The results revealed negative correlations between liver steatosis and pgRNA, HBV DNA, HBsAg, and HBeAg (pgRNA: r =  − 0.529, *P* < 0.001; HBV DNA: r =  − 0.456, *P* < 0.001; HBsAg: r =  − 0.465; *P* < 0.001; HBeAg: r =  − 0.339, *P* < 0.001) (Fig. 1). As the severity of liver steatosis increased, there was a corresponding decrease in the levels of pgRNA, HBV DNA, HBsAg, and HBeAg.

In liver steatosis grades S1, S2, and S3, a consistent descending trend was observed for the levels of pgRNA, HBV DNA, HBsAg, and the positive rate of HBeAg (pgRNA: 3.51 vs. 2.93 vs. 2.72 log10 copies/mL, *P* < 0.001; HBV DNA: 4.04 vs. 3.53 vs. 2.73 log10 IU/mL, *P* < 0.001; HBsAg: 3.47 vs. 3.09 vs. 2.52 log10 IU/mL, *P* < 0.001; positive rate of HBeAg: 41.2% vs. 19.5% vs. 5.8%, *P* < 0.001) (Table 5).

**Table 5:** Hepatitis B virological comparison among different degrees of hepatic steatosis (S1, S2, S3)

Variables	S1 (n = 51)	S2 (n = 41)	S3 (n = 52)	*P* value
pgRNA (log_10_ copies/mL)	3.51 (3.19–5.15)	2.93 (2.24–3.42)	2.72 (2.05–3.04)	< 0.001*
HBV DNA (log_10_ IU/mL)	4.04 (3.39–5.09)	3.53 (3.31–4.33)	2.73 (1.71–3.53)	< 0.001*
HBsAg (log_10_ IU/mL)	3.47 (3.13–3.95)	3.09 (2.14–3.39)	2.52 (1.00–3.29)	< 0.001*
Positive rate of HBeAg, n (%)	21 (41.2)	8 (19.5)	3 (5.8)	< 0.001*

Data are expressed as median (IQR) or number (%)

*Statistically significant at *P* < 0.05

### Logistic regression analysis of pgRNA below the LLD in patients infected with HBV

In 272 patients with HBV infection, univariate logistic regression analysis revealed significant correlations between pgRNA below the LLD and HBV DNA, HBsAg, triacylglycerol (TG), and CAP values (*P* < 0.05). Subsequently, a multivariable logistic regression incorporating these indicators was conducted. The results indicated that HBV DNA (OR: 0.283, 95% CI 0.146–0.550, *P* < 0.001), HBsAg (OR: 0.300, 95% CI 0.148–0.610, *P* < 0.001), and CAP values (OR: 1.013, 95% CI 1.001–1.026, *P* = 0.038) independently influenced the presence of pgRNA below the LLD in chronic HBV infection (Table 6).

**Table 6:** Multivariate logistic regression analysis for pgRNA below the LLD in patients with chronic HBV infection

Variables	Univariate			Multivariate		
	OR	95% CI	*P* value	OR	95% CI	*P* value
Men	1.322	0.744–2.350	0.342			
Age	1.011	0.986–1.037	0.386			
Cirrhosis	1.669	0.765–3.644	0.198			
Hepatocellular carcinoma	1.874	0.605–5.807	0.277			
HBV DNA	0.197	0.110–0.354	< 0.001*	0.283	0.146–0.550	< 0.001*
HBsAg	0.148	0.093–0.237	< 0.001*	0.300	0.148–0.610	< 0.001*
HBeAg	0.137	0.017–1.115	0.063			
ALT	1.003	0.994–1.013	0.478			
AST	1.001	0.993–1.008	0.882			
ALP	1.002	0.995–1.010	0.549			
GGT	1.003	0.997–1.010	0.325			
TC	1.194	0.917–1.554	0.188			
TG	1.547	1.167–2.051	0.002*	1.142	0.647–2.017	0.647
BMI	0.934	0.832–1.047	0.242			
CAP values	1.021	1.013–1.029	< 0.001*	1.013	1.001–1.026	0.038*
LSM values	1.020	0.978–1.063	0.361			

*OR* Odds ratio, *CI* Confidence interval, *ALT* Alanine aminotransferase, *AST* Aspartate aminotransferase, *ALP* Alkaline phosphatase, *GGT* Gamma-glutamyltransferase, *TC* Total cholesterol, *TG* Triacylglycerol, *BMI* Body mass index, *CAP* Controlled attenuation parameter, *LSM* Liver stiffness measurement

*Statistically significant at *P* < 0.05

## Discussion

Globally, the coexistence of CHB and MAFLD poses a significant public health challenge, with biopsy-proven MAFLD prevalence ranging from 13.5 to 30% in patients with CHB [8–10]. Despite the extensive prevalence, the causal relationship between these conditions remains elusive. The intricate interplay of metabolic disorders and immune dysregulation in MAFLD adds complexity to the HBV-MAFLD interaction, potentially accelerating the progression of severe liver disease. This study investigated the association between liver steatosis and hepatitis B virology to delve deeper into the impact of MAFLD on HBV infection, investigating factors influencing pgRNA levels in patients infected with HBV. The key findings are summarized below: (1) The expression of HBV in patients with CHB and liver steatosis exhibited a significant decrease, particularly in the high-level HBV DNA cohort, with significant reductions in HBsAg and pgRNA levels; (2) Liver steatosis demonstrated a negative correlation with hepatitis B virology, with higher degrees of liver steatosis associated with lower expression of HBV DNA, pgRNA, HBsAg, and HBeAg; (3) Independent influencing factors for pgRNA below the LLD in patients with HBV infection including HBV DNA, HBsAg, and CAP values.

A 3-year longitudinal cohort study reported that patients with CHB and liver steatosis had a higher chance of achieving HBsAg loss, with the HBsAg clearance rate after the 3-year follow-up being three times greater than that of those with CHB and without liver steatosis. This finding suggests an increased potential for clinical cure in patients with CHB and liver steatosis [11]. In this cross-sectional study of 272 patients with CHB, the association between liver steatosis and hepatitis B virology was sought to be investigated. Our findings revealed diminished HBV DNA, pgRNA and HBsAg levels in the CHB + MAFLD group, and a significant decrease in HBeAg positivity rates. These results suggest that hepatic steatosis inhibits HBV replication. Hu et al. [3] corroborated this perspective through mice model experiments, asserting that the decline in HBV DNA levels in mice with hepatic steatosis was linked to hepatocyte metabolism, indicating a direct impact of hepatic steatosis on HBV replication. Furthermore, our study unveiled a significant negative correlation between liver steatosis and hepatitis B virology. Spearman correlation analysis revealed a negative association between the degree of hepatic steatosis and HBV DNA, pgRNA, HBsAg, and HBeAg. Patients with severe liver steatosis exhibited significantly lower levels of HBV DNA, pgRNA, and HBsAg compared to those with mild or moderate liver steatosis, indicating a marked decrease in HBV levels with increasing severity of hepatic steatosis. Consistent with our findings, a retrospective study of 3212 patients with CHB demonstrated a negative correlation between the degree of hepatic steatosis and the level of HBsAg in hepatocytes. In patients with hepatic steatosis > 5%, HBsAg levels in hepatocytes were significantly lower than those in patients with hepatic steatosis < 5% and without hepatic steatosis [12]. According to the present study, MAFLD may inhibit HBV through various mechanisms. Firstly, MAFLD inhibits HBV replication by modulating immune responses, activating NK cells and CD8 + T cells through the Toll-like receptor (TLR) pathway to accelerate HBV clearance [13, 14]. TLR4/myeloid differentiation factor 88 (MyD88), a common signaling pathway in MAFLD and CHB, is critical in inhibiting HBV replication. TLR4/MyD88 downregulates the expression of two transcription factors (hepatocyte nuclear factor 1-alpha and hepatocyte nuclear factor 4-alpha) during HBV replication, potentially explaining the reduction in HBV replication in patients with CHB and MAFLD [15]. Some scholars propose that hepatic steatosis inhibits HBV replication by enhancing T-cell responses [16]. Secondly, MAFLD may accelerate the induction of apoptosis and inhibit autophagy in HBV-infected cells, ultimately leading to the clearance of HBsAg and HBV DNA [17, 18]. In addition, it has been confirmed that miR-122 inhibits HBV transcription by reducing the expression of cyclin G1 [19], while the expression level of miR-122 was increased in patients with MAFLD [20], which ultimately leads to the reduction of HBV expression in patients with CHB combined with MAFLD. Finally, MAFLD-related metabolic disorders, such as insulin resistance, diminish HBV replication by suppressing the activity of peroxisome proliferator-activated receptor-gamma (PPAR-γ) coactivator one alpha [21, 22].

To further investigate the relationship between liver steatosis and HBsAg, HBeAg, and pgRNA in patients with similar viral loads, our study encompassed both high-level HBV DNA and low-level HBV DNA populations. The findings indicated that, in both groups, the levels of pgRNA and HBsAg in CHB + MAFLD were lower compared to the CHB group. However, in high-level HBV DNA patients, the reduction was more substantial for pgRNA (3.85 vs. 3.35 log10 copies/mL, *P* = 0.044) and HBsAg (3.85 vs. 3.59 log10 IU/mL, *P* = 0.033). These results confirmed that liver steatosis led to varying HBV replication reduction in high-level HBV DNA and low-level HBV DNA populations. Adiponectin, a downstream target gene of PPAR-γ known to upregulate HBV replication, experiences reduced levels in patients with hepatic steatosis [23, 24]. Mohamadkhani et al. [25] demonstrated a negative correlation between HBV DNA levels and adiponectin. These results showed that in the CHB + MAFLD group with higher viral loads, adiponectin levels were lower, ultimately contributing to the decline in HBV replication ability. Given the relatively high viral load in high-level HBV DNA patients in this study, the presence of hepatic steatosis aggravated HBV clearance, suppressing HBV replication.

In recent years, accumulating evidence suggests that achieving HBV DNA below the LLD is not a sufficient endpoint for discontinuing antiviral therapy [26, 27]. As a precursor of HBV RNA, pgRNA provides insight into the transcriptional activity of cccDNA in hepatocytes [28]. Distinguished from HBsAg and HBV DNA, serum pgRNA exclusively originates from cccDNA, rendering it a valuable criterion for determining a complete virological response to antiviral therapy and a predictive indicator for virological recurrence post-discontinuation [29, 30]. The conversion to negative pgRNA, synonymous with cccDNA depletion or transcriptional silencing [31], signifies increased accessibility to clinical cures for CHB. This study employed multivariate logistic regression analysis to explore further the relevant influencing factors of pgRNA below the LLD in patients infected with HBV. The results revealed that HBV DNA, HBsAg, and CAP values emerged as independent influencing factors for pgRNA negativity in CHB. Therefore, lower HBsAg and HBV DNA levels, coupled with higher CAP values, indicated a higher likelihood for patients with CHB to achieve pgRNA below the LLD, reaffirming the supportive role of liver steatosis in HBV clearance.

This study carries certain limitations. Firstly, a cross-sectional analysis was conducted, lacking a dynamic observation of hepatic steatosis’s influence on HBV through longitudinal follow-up. Future studies will address this by implementing longitudinal follow-up to gauge the impact of liver steatosis on antiviral therapy for CHB. Secondly, this study lacked liver pathological biopsy, relying solely on the serological analysis of HBV expression. To provide a more comprehensive understanding of hepatic steatosis’s impact on hepatitis B virology, disease progression, and prognosis, a multicenter, large-sample prospective cohort study is warranted.

## Conclusion

Our study revealed reduced HBV expression among patients with CHB and hepatic steatosis, particularly significant in high-level HBV DNA patients with a higher viral load. Furthermore, a negative correlation was identified between hepatic steatosis and hepatitis B virology, observing a gradual decline in the levels of HBsAg, HBV DNA, and pgRNA and the positive rate of HBeAg with worsening hepatic steatosis. Finally, our findings highlighted HBV DNA, HBsAg, and CAP values as independent factors influencing pgRNA levels below the LLD in patients infected with HBV. These insights could hold clinical significance for the future monitoring and treatment of patients with CHB and MAFLD. Subsequent research, encompassing primary and clinical studies, is needed to explore further the impact of hepatic steatosis on the natural course of HBV and patient prognosis.

## Data Availability

The datasets generated and/or analysed during the current study are not publicly available but are available from the corresponding author upon reasonable request.

## Abbreviations

CHB: Chronic hepatitis B
MAFLD: Metabolic dysfunction-associated fatty liver disease
HBV: Hepatitis B virus
HBsAg: Hepatitis B surface antigen
pgRNA: Pregenomic RNA
HBeAg: Hepatitis B e antigen
OR: Odds ratio
ALT: Alanine aminotransferase
cccDNA: Covalently closed circular DNA
SAT: Simultaneous amplification testing
S/Co: Signal-to-cutoff ratio
LLD: Lower limit of detection
CAP: Controlled attenuation parameter
LSM: Liver stiffness measurement
IQR: Interquartile range
95% CI: 95% Confidence interval
BMI: Body mass index
AST: Aspartate aminotransferase
ALP: Alkaline phosphatase
GGT: Gamma-glutamyltransferase
TC: Total cholesterol
TG: Triacylglycerol
TLR4: Toll-like receptor 4
MyD88: Myeloid differentiation factor 88
PPAR-γ: Peroxisome proliferator-activated receptor-gamma
